# Molecular Characteristics, Antigenicity, Pathogenicity, and Zoonotic Potential of a H3N2 Canine Influenza Virus Currently Circulating in South China

**DOI:** 10.3389/fmicb.2021.628979

**Published:** 2021-03-09

**Authors:** Meihua Wu, Rongsheng Su, Yongxia Gu, Yanan Yu, Shuo Li, Huapeng Sun, Liangqi Pan, Xinxin Cui, Xuhui Zhu, Qingzhou Yang, Yanwei Liu, Fengxiang Xu, Mingliang Li, Yang Liu, Xiaoyun Qu, Jie Wu, Ming Liao, Hailiang Sun

**Affiliations:** ^1^College of Veterinary Medicine, South China Agricultural University, Guangzhou, China; ^2^Key Laboratory of Zoonosis, Ministry of Agriculture and Rural Affairs, Guangzhou, China; ^3^Guangdong Laboratory for Lingnan Modern Agriculture, Guangzhou, China; ^4^National and Regional Joint Engineering Laboratory for Medicament of Zoonosis Prevention and Control, Guangzhou, China; ^5^Key Laboratory of Zoonosis Control and Prevention of Guangdong Province, Guangzhou, China; ^6^Guangdong Provincial Center for Disease Control and Prevention, Guangzhou, China

**Keywords:** H3N2 canine influenza virus, HA stability, antigenicity, pathogenicity, guinea pig

## Abstract

Canine influenza viruses (CIVs) could be a source of influenza viruses which infect humans because canine are important companion pets. To assess the potential risk of H3N2 CIVs currently circulating in southern China to public health, biological characteristics of A/canine/Guangdong/DY1/2019 (CADY1/2019) were detected. CADY1/2019 bound to both avian-type and human-type receptors. CADY1/2019 had a similar pH value for HA protein fusion to human viruses, but its antigenicity was obviously different from those of current human H3N2 influenza viruses (IVs) or the vaccine strains recommended in the North hemisphere. CADY1/2019 effectively replicated in the respiratory tract and was transmitted by physical contact among guinea pigs. Compared to human H3N2 IV, CADY1/2019 exhibited higher replication in MDCK, A549, 3D4/21, ST, and PK15 cells. Sequence analysis indicated that CADY1/2019 is an avian-origin virus, and belongs to the novel clade and has acquired many adaptation mutations to infect other mammals, including human. Taken together, currently circulating H3N2 CIVs have a zoonotic potential, and there is a need for strengthening surveillance and monitoring of their pathogenicity.

## Introduction

The Influenza A viruses (IAVs) belonging to the genus orthomyxovirus (Alexander and Brown, 2000) can cause endemics or pandemics (Webster et al., 1992). IAVs can infect broad-spectrum animals, including birds, human, swine, seals, whales, horses, dogs, and cats (Webster et al., 1992; Keawcharoen et al., 2004; Peiris et al., 2007; Sun et al., 2011; Yoon et al., 2014). A critical feature of ecology and epidemiology of IAVs is interspecies transmission (Webster, 1998; Webby et al., 2004). Equine-origin H3N8 canine influenza virus (CIV) was reported in America in 2004 (Crawford et al., 2005). Avian-origin H3N2 CIV was first isolated in South Korea in 2007. Besides, 2009 pandemic H1N1 virus (pdm/09), avian and swine IAVs were also reported infection in canine. Furthermore, α-2, 3, and α-2,6 gal sialic acids distribute on dog’s tracheal epithelial cells (Daly et al., 2008; Muranaka et al., 2011) and canines are considered as potential mixing vessels for reassortment between IVs (Chen et al., 2018). To some extent, CIVs pose a threat to public health.

H3N8 and H3N2 are the major subtypes of CIVs that are circulating in canines worldwide. H3N8 CIV was first isolated in racing greyhounds in Florida in 2004 (Crawford et al., 2005). Subsequently, H3N8 CIVs were reported to circulate in America, Canada, Britain, and Australia (Daly et al., 2008; Kruth et al., 2008; Kirkland et al., 2010; Rivailler et al., 2010; Crispe et al., 2011). In 2007, an avian-origin H3N2 CIV was first isolated in Korea (Song et al., 2008), which could be traced back to 2006 in China (Li et al., 2010). Since then, H3N2 CIVs have spread globally and circulated in Korea, China, Thailand, the United States, and Canada (Su et al., 2013; Bunpapong et al., 2014; Lee et al., 2016; Voorhees et al., 2018; Xu et al., 2020). Besides H3N2 and H3N8 CIVs, dogs were also reported to be infected with H5N1, H9N2, H6N1, and recombinant H5N2 avian influenza viruses (AIVs) (Songserm et al., 2006; Zhan et al., 2012; Sun et al., 2013; Lin et al., 2015), H1N1 swine influenza viruses (SIV) (Chen et al., 2018), H3N2 IAVs (Chen et al., 2015), and 2009 pandemic H1N1 virus (pdm/09) (Dundon et al., 2010; Su et al., 2014). Notably, reassortment viruses between H3N2 CIVs and pdm/09 viruses appeared in 2010–2012, and recombinants between H3N2 CIVs and swine-origin H1N1 viruses emerged in 2013–2015 (Song et al., 2012; Moon et al., 2015; Na et al., 2015; Chen et al., 2018). Also, CIVs can break through the species-barrier to infect cats (Song et al., 2011). These findings increase the concern about the zoonotic potential of CIVs.

H3N2 CIVs replicate in the respiratory tract of dogs and can be transmitted among them through physical contact or airborne droplet. H3N2 CIVs could transmit from experimentally infected dogs to contact-exposed dogs, causing these dogs to exhibit clinical signs (Song et al., 2009). Besides contact transmission, A/canine/Zhejiang/01/2010 (H3N2) (ZJ0110) could infect 6 month-old beagles by airborne transmission, causing severe respiratory syndrome (Teng et al., 2013). A/canine/Korea/AS-01/2009 (AS-01/09), A/canine/Korea/AS-05/2012 (AS-05/12), and A/canine/Korea/AS-11/2013 (AS-11/13) readily droplet-transmitted between dogs, of which, AS-05/12 induced more severe clinical diseases and fatalities in dogs compared with AS-01/09 (Lee et al., 2018). Besides dogs, H3N2 CIVs can infect other mammals. ZJ0110 H3N2 CIV could replicate in the upper respiratory tracts of mice and guinea pigs, and virus titers were comparable to those of dogs (Teng et al., 2013). CIV could induce influenza-like clinical signs, viral shedding, and serological responses in infected cats and ferrets, and transmit to naïve cats and ferrets via direct contact with infected animals of the same species (Kim et al., 2013). AS-05/12 exhibited higher viral shedding titers and airborne droplet transmission among ferrets (Lee et al., 2018). Interspecies transmission (dogs to cats) of CIV H3N2 via an airborne route was observed in experimentally infected animals (Kim et al., 2013). Also, cats infected with CIV H3N2 were reported in Korea (Song et al., 2011). These findings suggest that H3N2 CIVs have the potential to infect other mammals.

Dogs are susceptible to IAVs and have the potential to be a new mixing vessel for IAVs to generate a novel pandemic virus. Here we assess the zoonotic potential of A/canine/Guangdong/DY1/2019 (CADY1/2019), a H3N2 CIV which is circulating in dogs in southern China. The aims of this study were: (1) to characterize the virus’ molecular and antigenic features, (2) to assess the receptor-binding ability of the virus, (3) to detect the pH value of activation and inactivation for the virus, (4) to assess the virus’ replication in mammalian cells, and (5) to illustrate the virus’ infectivity and transmission in guinea pigs.

## Materials and Methods

### Cells and Viruses

Madin-Darby canine kidney (MDCK) cells, human lung adenocarcinoma epithelial (A549) cells, African green monkey kidney (Vero) cells, porcine kidney (PK-15) cells, porcine alveolar macrophage (3D4/21) cells, and swine testicle (ST) cells were stored in our laboratory. Human bronchial epithelioid (HBE) cells were purchased from OTWO biotech incorporation (Shenzhen city, China). All cells were cultured in Dulbecco’s modified Eagle’s medium (DMEM) (Gibco, Grand Island, NY, United States) containing 10% fetal bovine serum (Gibco, Grand Island, NY, United States) and 1% penicillin-streptomycin (PS) (BI, Kibbutz Beit Haemek, Israel). The CADY1/2019 was isolated from the nasal swab from a dog with influenza-like symptom in Guangdong province in 2019 and was propagated in MDCK cells and stored at −80°C. The human H3N2 seasonal influenza virus, A/Guangdong/47/2020 (47/2020), being isolated and purified in Guangdong Provincial Center for Disease Control and Prevention, was used as control. The human H3N2 seasonal influenza virus, A/Guangdong/1194/2019 (1194/2019) that was isolated and purified in Guangdong Provincial Center for Disease Control and Prevention, was used for viral antigen analysis.

### Molecular and Genetic Analysis

Total RNA was extracted from the suspension of cells culture, and RT-PCR was performed using Uni12 (AGCAAAAGCAGG). The eight viral segments were amplified by PCR using specific primers (Hoffmann et al., 2001). After electrophoresis, PCR products were purified using the Gel Extraction Kit D2500 (Omega Bio-Tek, Guangzhou, China), and were then sequenced by Tianyihuiyuan Biotechnology Co. in Guangzhou city, Guangdong province. The sequences were spliced using Seqman software of Lasergene (Version 7.1). The phylogenetic trees of CADY1/2019 were constructed using of MEGA7.0 software.

### Receptor-Binding Assay

The receptor binding characteristic of CADY1/2019 was detected by a solid-phase binding assay as previously reported (Sun et al., 2019). 47/2020 was used as control. Briefly, polystyrene Universal-Bind microplates (Corning, New York, United States) were coated with 1 μg streptavidin (PuriMag Biotech, Xiamen, China) in 99 μL phosphate citrate buffer at 37°C for 12–24 h until dry. After being washed three times with PBST (phosphate-buffered saline containing 0.05% Tween-20), the plates were incubated with α-2, 3-siaylglycopolymer or α-2, 6-siaylglycopolymer (GlycoTech, Inc., Gaithersburg, Maryland, United States) at 4°C for 24 h. The concentrations of siaylglycopolymer were twofold diluted to 0.78–100 ng in 100 μL PBS. After that, the plates were washed with PBS for three times and then incubated with virus in a titer of 64 HA units at 4°C. Twelve hours later, guinea pig sera against CADY1/2019 and a monoclonal antibody against H3 subtype AIV (Zoonogen, Beijing, China) were added in the plates, respectively, after being washed with PBST for three times. After being inoculated at 4°C for 5 h, the plates were washed three times with PBST and incubated with horseradish peroxidase (HRP)-conjugated goat anti-guinea pig antibody (Beijing Bioss Biological Technology, Beijing, China) or HRP-conjugated goat anti-mouse antibody (Bioworld Technology, Nanjing, China) at a concentration of 100 ng/mL at 4°C for 2 h. Next, the plates were washed three times with PBST and incubated with TMB (3, 3′, 5, 5′-Tetramethylbenzidine) (Solarbio, Beijing, China) at room temperature. Then the reaction was terminated by ELISA stop solution (Solarbio, Beijing, China) 5 min later. Then the optical density (OD) values at 450 nm were determined in a plate reader.

### HA Acid Stability

The HA activation pH value of CADY1/2019 and 47/2020 were detected by syncytia assay described by a previous study (Reed et al., 2010). Briefly, when the confluence of Vero cells was about 90%, cells were infected with the virus at a multiplicity of infection (MOI) of 3. At 16 h post-infection (hpi), cells were incubated with TPCK-treated trypsin for 5 min, incubated with pH-adjusted PBS buffers for 5–10 min, neutralized, and finally cultured in DMEM containing 10% FBS for 3 h at 37°C. Subsequently, cells were fixed with absolute methanol and stained with haematoxylin and eosin (HE) for microscopy. To assess the resistance of viruses against inactivation caused by acid *in vitro*, 10 μL of virus stocks were diluted in 990 μL of pH-adjusted PBS solutions and incubated for 1 h at 37°C. Then, the infectivity of the virus was detected in MDCK cells. The infectivity curves of viruses were fitted to an asymmetric (5-parameter) regression model. The pH_50_ values were determined as the point at which viruses lose their 50% infectivity.

### Antigenic Analysis of CADY1/2019

Guinea pig serum samples against CADY1/2019, 1194/2019, or 47/2020 were treated with receptor-destroying enzyme (RDE) (Denka Seiken Co., Tokyo, Japan) at 37°C for 18–20 h. Then, serum samples were bathed at 56°C for 30 min. The treated serum samples were 10-fold diluted with PBS. Hemagglutination inhibition (HI) assay was performed with 0.5% turkey erythrocytes, as described (Hirst, 1942). The treated serum samples were serially twofold diluted with Reduced Serum Medium (Opti-MEM medium) (Sigma-Aldrich, St. Louis, MO) and were then incubated with viruses with 100 TCID_50_ at 37°C for 2 h. Then, the mixture was added into MDCK cells and incubated at 37°C for 2 h. Next, the inoculation was replaced by Opti-MEM medium containing TPCK-treated trypsin with a concentration of 1 μg/mL and cells were cultured in an incubator at 37°C with 5% CO_2_ for 72 h. Microneutralization (MN) titers were detected and expressed as the geometric mean titer.

### Replication of Viruses in Mammalian Cells

The *in vitro* replication of CADY1/2019 was characterized in MDCK, A549, PK-15, 3D4/21, ST, and HBE cells. A human H3N2 influenza virus, 47/2020, was used as a control. When the confluence of the cells in 12-well plates was about 90%, MDCK cells were infected with CADY1/2019 or 47/2020 at a MOI of 0.001; the other cells were inoculated with CADY1/2019 or 47/2020 at a MOI of 0.01. At 2 h post-infection (hpi), inoculants were discarded, and cells were washed with PBS for twice and then cultured in Opti-MEM I Reduced Serum Medium (Sigma-Aldrich, St. Louis, MO) with 1.0 μg/mL TPCK-treated trypsin and 1% penicillin and streptomycin. Supernatants of cells were collected at 12, 24, 36, 48, 60, and 72 hpi and were then titrated in MDCK cells.

### Pathogenicity in Guinea Pigs

Nineteen female, 6 week-old, SPF Hartley strain guinea pigs were randomly divided into five groups to detect viral replication and transmission. Of which, under anaesthesia, three guinea pigs were intranasally inoculated with PBS in a volume of 300 μL as a negative control. To detect viral replication in guinea pigs, four guinea pigs were equally divided into two groups and were intranasally inoculated with CADY1/2019 or 47/2020 at a dose of 10^6^ TCID_50_ in 300 μL under anaesthesia. Two infected guinea pigs and one control guinea pig were necropsied at 3 dpi, and turbinate, trachea, and lung were collected. The viral titers in tissues of guinea pigs were titrated in MDCK cells.

To detect viral transmission, six guinea pigs were equally divided into two groups and were intranasally inoculated with CADY1/2019 or 47/2020 at a dose of 10^6^ TCID_50_ in 300 μL under anaesthesia. At 1 dpi, three naïve guinea pigs were co-housed with three infected guinea pigs in the same cage. The nasal washes of guinea pigs were collected at 2, 4, 6, 8, and 10 dpi and were titrated in MDCK cells. The sera were collected at 21 dpi for HI titration.

### Statistical Analysis

Statistical analysis was performed using Prism 7.0 software (GraphPad, La Jolla, CA). The data between the groups were compared using *t*-tests. A *p*-value < 0.05 was considered to be significant. ^∗^ indicates *P* < 0.05, ^∗∗^ indicates *P* < 0.01, ^∗∗∗^ indicates *P* < 0.001.

### Ethics Statements

This study was carried out in ABSL-2 facilities in compliance with approved protocols by the biosafety committee of South China Agricultural University. The handling of guinea pigs was conducted in compliance with the approved guidelines of the Experimental Animal Administration and Ethics Committee of South China Agriculture University (SCAUABSL2020-005; 5 May, 2020).

## Results

### CADY1/2019 Was an Avian-Origin Virus and Formed a Novel Subclade Together With Current CIVs

To analyze the homology and phylogenetic of the virus, the entire genome of CADY1/2019 was amplified, sequenced, and submitted to GenBank at NCBI, and the accession numbers are MW126982–MW126989. Except for the NS gene, the other genes of CADY1/2019 shared the highest degree (99.12–99.87%) with the corresponding gene of A/canine/Guangdong/3/2018. The NS gene shared the highest similarity (99.40%) with those of A/canine/China/Shanghai-0103-2045/2019 and A/canine/Guangdong/2/2018, and exhibited higher homology (99.28%) with that of A/canine/Guangdong/3/2018 (Supplementary Table S1).

**FIGURE 1:**
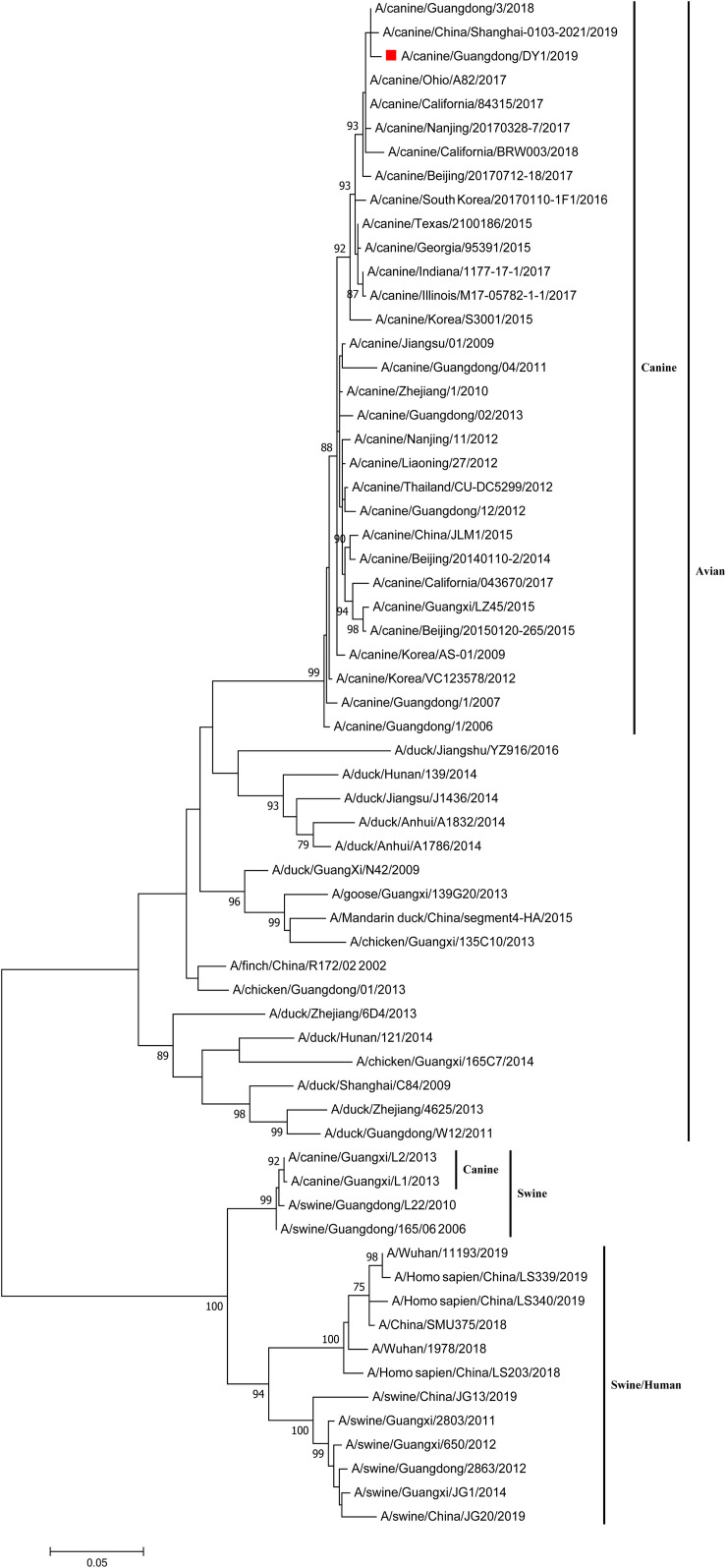
Phylogenetic analysis of HA gene of CADY1/2019. Phylogenetic trees for whole gene segments of CADY1/2019 created with 1000 bootstrap replicates using MEGA 7.0 software. Solid red square indicated CADY1/2019 in each phylogenetic tree. Phylogenetic trees of HA genes based on nucleotides (nt) 30–1,730.

**FIGURE 2:**
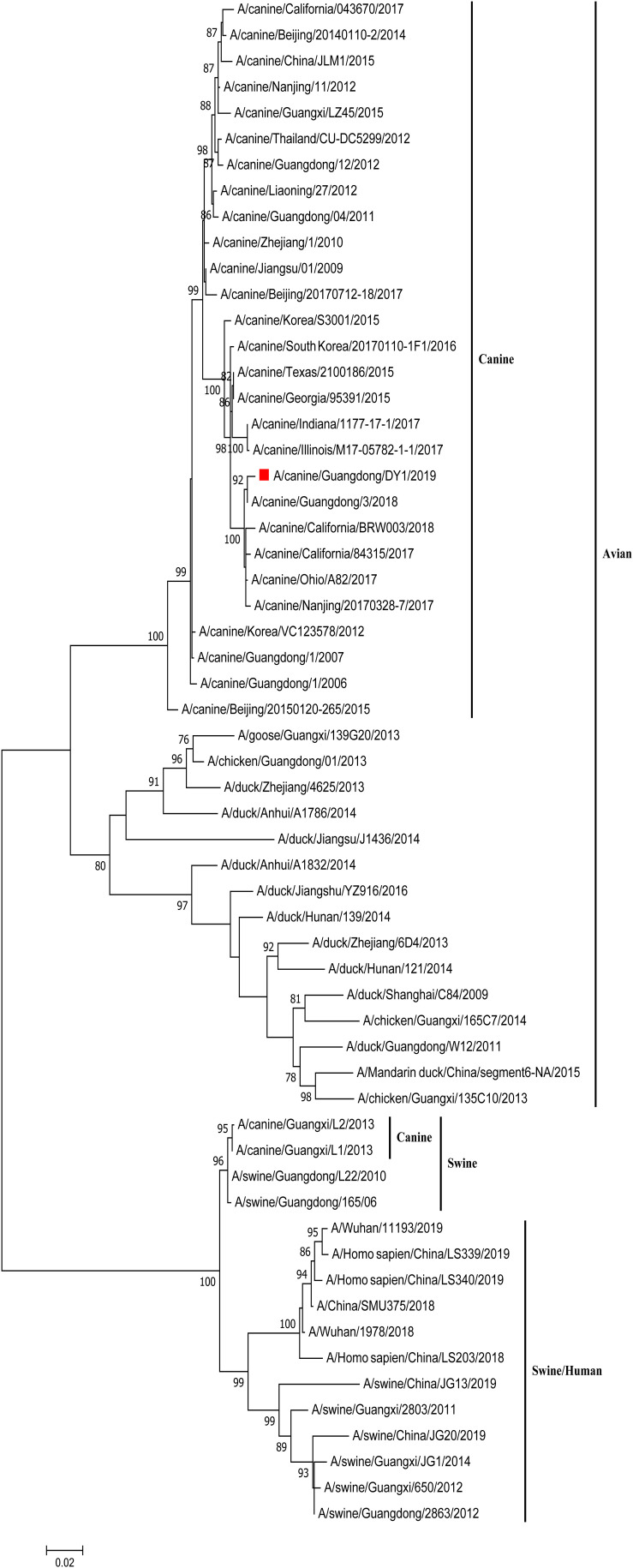
Phylogenetic analysis of NA gene of CADY1/2019. Phylogenetic trees for whole gene segments of CADY1/2019 created with 1000 bootstrap replicates using MEGA 7.0 software. Solid red square indicated CADY1/2019 in each phylogenetic tree. Phylogenetic trees of NA genes based on nt 20–1,429.

The eight phylogenetic trees of CADY1/2019 were constructed by Maximum Likelihood method using the MEGA 7.0 software. The phylogenetic tree of each gene segment was divided into three subgroups: canine and avian lineage, swine lineage, and swine/human lineage. The eight genes of CADY1/2019 were clustered into the canine and avian lineage and were close with those of CIVs circulating in China and the United States in recent years. This indicated that CADY1/2019 was an avian-origin CIV (Figures 1, 2 and Supplementary Figures S1–S6). All genes of CADY1/2019 were close to those of A/canine/Nanjing/20170328-7/2017 which belonged to a novel subclade emerged in 2016. And viruses in such novel subclade were reported phylogenetically and antigenically different from previously circulating CIV strains (Lyu et al., 2019).

### CADY1/2019 Acquired Adaptation Mutations to Infect Mammals

To better understand the molecular characteristics of CADY1/2019, its whole genomes were analyzed. CADY1/2019 possessed 214I and 159N in HA protein which favors IAVs to bind the human-like receptor. CADY1/2019 carried 222L in the HA protein, 389R in the PB2 protein, 3V in the PB1 protein, and 409S in the PA protein, which can enhance the replication of AIVs in mammalian cells. In addition, CADY1/2019 posed the amino acids 89V and 309D in PB2 protein; 622G in PB1 protein; 66S in PB1-F2 protein; 224S in PA protein; 42S, 106M, 103F, and 106M in NS1 protein; and 30D and 215A in M1 protein, which were associated with increasing replication and virulence of AIVs in mammalian hosts (Table 1).

**TABLE 1:** The major amino acids of CADY1/2019 that may affect functions.

Gene	AA substitution	Function	Amino acid(s)	References
HA (H3 numbering)	V214I	Enhanced binding affinity of H5N1 IAV for α-2,6 SA	I	Watanabe et al., 2011
	S159N	Enhanced binding affinity of H5N1 IAV for α-2,6 SA	N	Wang et al., 2010
	W222L	Increased infectivity and replication efficiency of H3N2 CIV in mammalian cells	L	Yang et al., 2013
PB2	K389R	Enhanced polymerase activity and virus growth capacity of H7N9 IAV in human and mammalian cells	R	Hu et al., 2017
	L89V + G309D	Enhanced polymerase activity of H5N1 IAV in mammalian cell and increased virulence of H5N1 IAV in mice	V + D	Li et al., 2009
PB1	D3V	Enhanced polymerase activity and viral replication of H5N1 IAV in avian and mammalian cells	V	Elgendy et al., 2017
	D622G	Enhanced polymerase activity and virulence of H5N1 IAV in mice	G	Feng et al., 2016
PB1-F2	N66S	Enhanced replication, virulence and antiviral response of H5N1 IAV in mice	S	Conenello et al., 2007; Schmolke et al., 2011
PA	P224S	Increased the virulence of pdmH1N1 in mice	S	Sun et al., 2014
	N409S	Enhanced polymerase activity and replication of H7N9 IAV in A549 cells	S	Yamayoshi et al., 2014
NS1	P42S	Increased virulence and decreased antiviral response of H5N1 IAV in mice	S	Jiao et al., 2008
	I106M	Enhance the replication and pathogenicity of H7N9 IAV in mice	M	Ayllon et al., 2014
	L103F + I106M	Increased replication and virulence of H5N1 IAV in mice	F + M	Spesock et al., 2011
M1	N30D	Enhance virulence of H5N1 IAV in mice	D	Fan et al., 2009
	T215A	Enhance virulence of H5N1 IAV in mice	A	Fan et al., 2009

### CADY1/2019 Bound to Both Avian-Type and Human-Type Receptors

**FIGURE 3:**
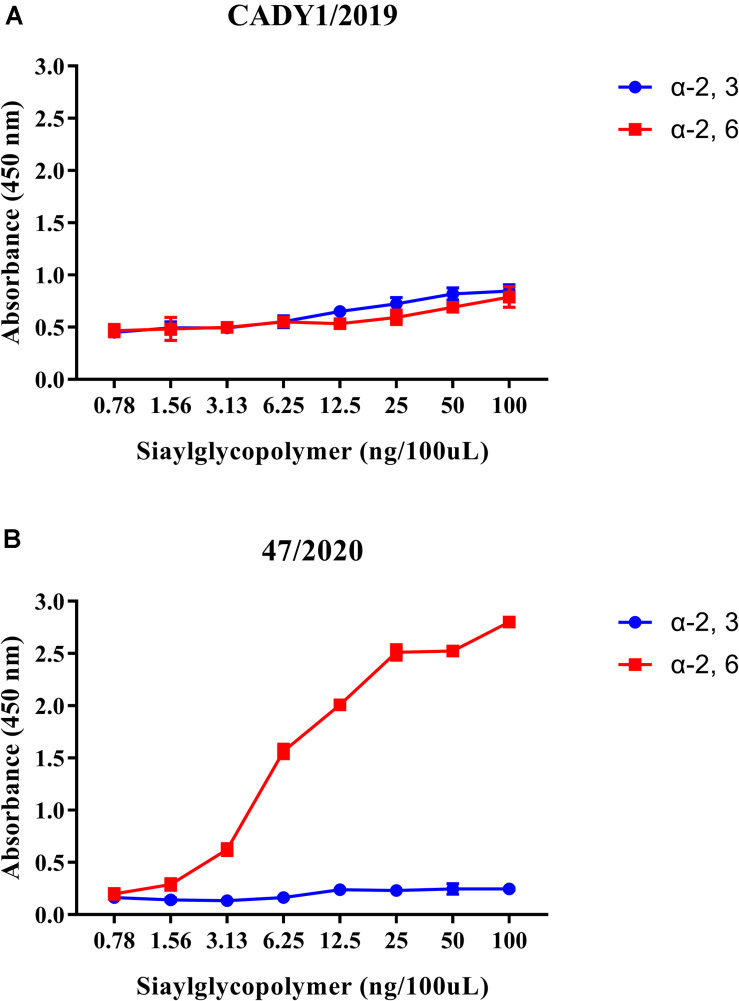
Receptor-binding ability of CADY1/2019 **(A)** and 47/2020 **(B)**. The binding ability of the viruses to two different biotinylated glycans (α-2, 3-siaylglycopolymer, colored in blue, α-2, 6-siaylglycopolymer, colored in red) were detected. The antibody used for the receptor-binding ability of CADY1/2019 was guinea pig sera anti CADY1/2019. The antibody used for the receptor-binding ability of 47/2020 was a monoclonal antibody against H3 subtype AIV that reacts with 47/2020. Every experiment was conducted twice, expressed as mean ± SD.

To better assess the zoonotic potential of CADY1/2019, the receptor-binding affinity of the virus was detected by a solid-phase binding assay. The result showed that CADY1/2019 bound to both α-2, 3-siaylglycopolymer (Avian receptor), and α-2, 6-siaylglycopolymer (Human receptor) (Figure 3A). 47/2020 strongly and exclusively bound to α-2, 6-siaylglycopolymer (Human receptor) (Figure 3B).

### CADY1/2019 Shared Similar Activation pH of HA Protein With 47/2020

To better assess the zoonotic potential of CADY1/2019, the stability of HA protein was measured by syncytia assay for three times. Human virus 47/2020 was used as the control. The results showed that HA activation pH value of CADY1/2019 and 47/2020 were 5.5 and 5.4, respectively. HA inactivation pH value of CADY1/2019 and 47/2020 were 5.40 and 5.28, respectively. CADY1/2019 posed similar HA stability with 47/2020. There was an approximate 0.1 units discrepancy between them in activation and inactivation pH value, respectively.

### Antigenicity of CADY1/2019 Was Different From Current Human H3N2 IAVs

To illustrate the antigenicity relationship between CADY1/2019 and current human H3N2 IAVs, HI and MN assays were performed. 1194/2019 and 47/2020 carry the same amino acids at key antigenic sites: 145, 155, 156, 158, 159, 189, and 193 in the HA protein as vaccine candidate viruses recommended by the WHO for the northern hemisphere during 2017–2019 and 2020–2021, respectively. Compared to the human H3N2 IAVs, CADY1/2019 contained N, K, G, N, and Q at positions 145, 156, 158, 159, and 189, respectively (Table 2). The HI titers of CADY1/2019, 1194/2019, and 47/2020 sera were 1:1613, 1:1028, and 1:5120, respectively. The neutralization titers of CADY1/2019, 1194/2019, and 47/2020 sera were 1:113, 1:135, and 1:95, respectively. The sera against CADY1/2019 did not react with 1194/2019 or 47/2020 virus, and vice versa. This indicated that the antigenicity of CADY1/2019 was completely different from those of recent human H3N2 IAVs (Table 3).

**TABLE 2:** Key antigen residues of H3N2 viruses.

Strains	145	155	156	158	159	189	193
CADY1/2019	N	T	K	G	N	Q	S
A/canine/Guangdong/3/2018^*a*^	N	T	K	G	N	Q	S
A/Hong Kong/4801/2014b	S	T	H	N	Y	K	F
A/Singapore/INFIMH-16-0019/2016^*b*^	S	T	H	N	Y	K	F
A/Kansas/14/2017^*b*^	S	T	H	N	S	K	S
A/Hong Kong/2671/2019^*b*^	S	T	H	N	Y	K	S
A/Hong Kong/45/2019^*b*^	S	T	H	N	Y	K	S
A/Guangdong/SKLRD01/2017^*c*^	S	T	H	N	Y	K	F
A/Guangdong/YueFang277/2017^*c*^	S	T	H	N	Y	N	S
A/Hong_Kong/2671/2019^*c*^	S	T	H	N	Y	K	S
1194/2019^*c*^	S	T	H	N	Y	K	F
47/2020^*c*^	S	T	H	N	Y	K	S

**^*a*^H3N2 CIV of which the HA gene shared highest degree with that of CADY1/2019 at nucleotide level. ^*b*^Vaccine candidate viruses recommended by WHO for using during 2017–2021 northern hemisphere influenza season. ^*c*^Human H3N2 strains recently isolated from southern China.**

**TABLE 3:** The antigenicity relationship between CADY1/2019 and human H3N2 influenza viruses.

Virus	Guinea pig sera					
	CADY1/2019		47/2020		1194/2019	
	HI^*a*^	MN^*b*^	HI	MN	HI	MN
CADY1/2019	1,613	113	<10	<10	<10	<10
1194/2019	<10	<10	50	14	1,028	135
47/2020	<10	<10	5,120	95	202	<10

**^*a*^HI, hemagglutination inhibition. ^*b*^MN, microneutralization; HI and MN titers were expressed at geometric mean titer.**

### CADY1/2019 Effectively Replicated in Mammalian Cells

**FIGURE 4:**
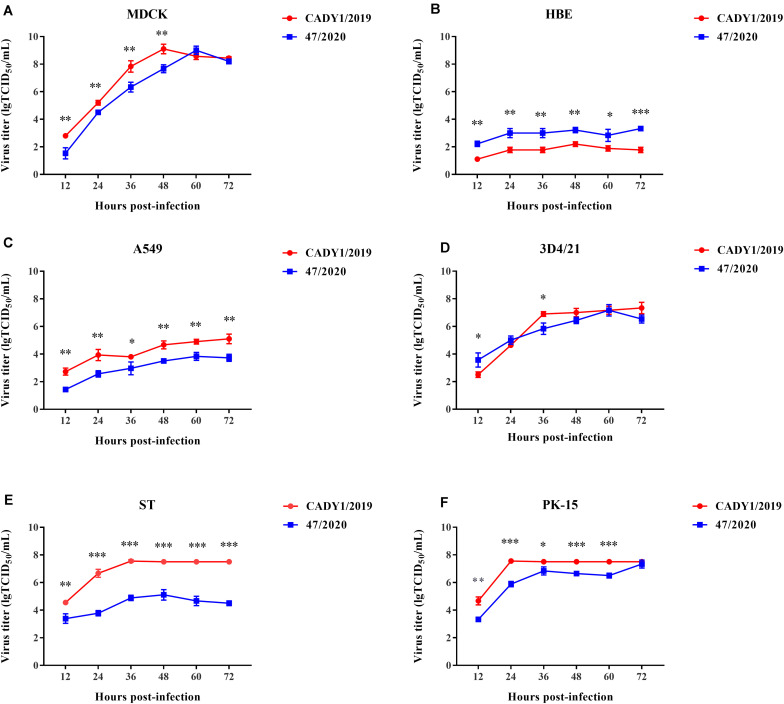
Growth kinetics of viruses in mammalian cells. Excepting MDCK cells **(A)** were infected with viruses at a MOI of 0.001, HBE **(B)**, A549 **(C)**, 3D4/21 **(D)**, ST **(E)**, and PK-15 cells **(F)** were inoculated with viruses at a MOI of 0.01. The supernatants of cells were collected at 12, 24, 36, 48, 60, and 72 hpi and titrated in MDCK cells. The experiments were repeated for three times and the viral titer at each time point was expressed as mean ± standard deviation. Statistical analysis was performed using Prism 7.0 software (GraphPad, La Jolla, CA). ^∗^ indicates *P* < 0.05, ^∗∗^ indicates *P* < 0.01, ^∗∗∗^ indicates *P* < 0.001.

To evaluate the replication capability of CADY1/2019 *in vitro*, the growth kinetics of the virus in six cells were detected. Human virus, 47/2020, was used as control. CADY1/2019 efficiently replicated in MDCK cells, 3D4/21 cells, ST cells, PK-15 cells, and A549 cells. CADY1/2019 exhibited higher replication ability in MDCK cells than in other cells (*p* < 0.01), and reached a peak titer of 9.10 ± 0.346 lgTCID_50_/mL at 48 hpi. In 3D4/21 cells, CADY1/2019 reached the viral peak with a titer of 7.33 ± 0.416 lgTCID_50_/mL at 72 hpi. In ST and PK-15 cells, the viral peak of CADY1/2019 (7.56 ± 0.098 lgTCID_50_/mL) appeared at 32 and 24 hpi, respectively. In A549 cells, the viral peak (with a titer of 5.10 ± 0.346 lgTCID_50_/mL) appeared at 72 hpi. In HBE cells, CADY1/2019 reached the maximum titer (2.2 ± 0.173 lgTCID_50_/mL) at 48 hpi. There was no statistically significant difference between the peak titers of CADY1/2019 and 47/2020 in MDCK, PK-15, and 3D4/21 cells (*p* > 0.05). The peak titers of CADY1/2019 in ST and A549 cells were significantly higher than that of 47/2020 (*p* < 0.001 and *p* < 0.01, respectively), while the peak titer of 47/2020 in HBE cells was higher than that of CADY1/2019 (*p* < 0.001) (Figure 4).

### CADY1/2019 Transmitted Among Guinea Pigs Through Direct Contact

**FIGURE 5:**
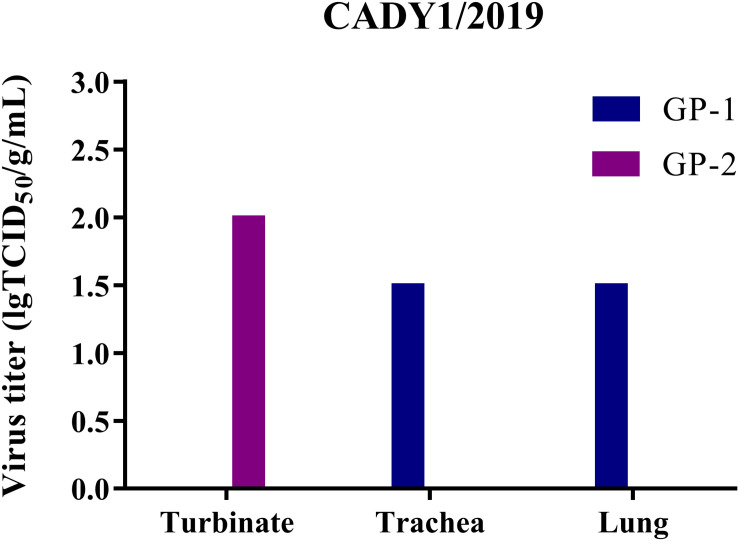
The virus titers in different tissues of guinea pigs in CADY1/2019 inoculation group. Four guinea pigs were inoculated intranasally at a dose of 10^6^ TCID_50_ of CADY1/2019 or 47/2020 in 300 μL. At 3 dpi, two infected guinea pigs in each virus experimental group were euthanized and turbinates, tracheas and lungs of them were collected for virus titration in MDCK cells. 47/2020 did not replicate in respiratory tissues of guinea pigs.

To detect the replication of CADY1/2019 *in vivo*, two guinea pigs were inoculated intranasally with 10^6^ TCID_50_ of the virus. At 3 dpi, viral titers in the respiratory tract were titrated in MDCK cells. Human H3N2 influenza virus 47/2020 was used for the control. The results showed that viral titers of CADY1/2019 in turbinate, trachea, and lung in the treatment group were 2.00, 1.50, and 1.50 lgTCID_50_/g/mL, respectively (Figure 5). The viral titers of 47/2020 in respiratory tissues of guinea pigs were undetectable. This indicated that compared to human influenza virus, CADY1/2019 replicated efficiently in both upper and lower respiratory tract of guinea pigs without prior adaptation.

**FIGURE 6:**
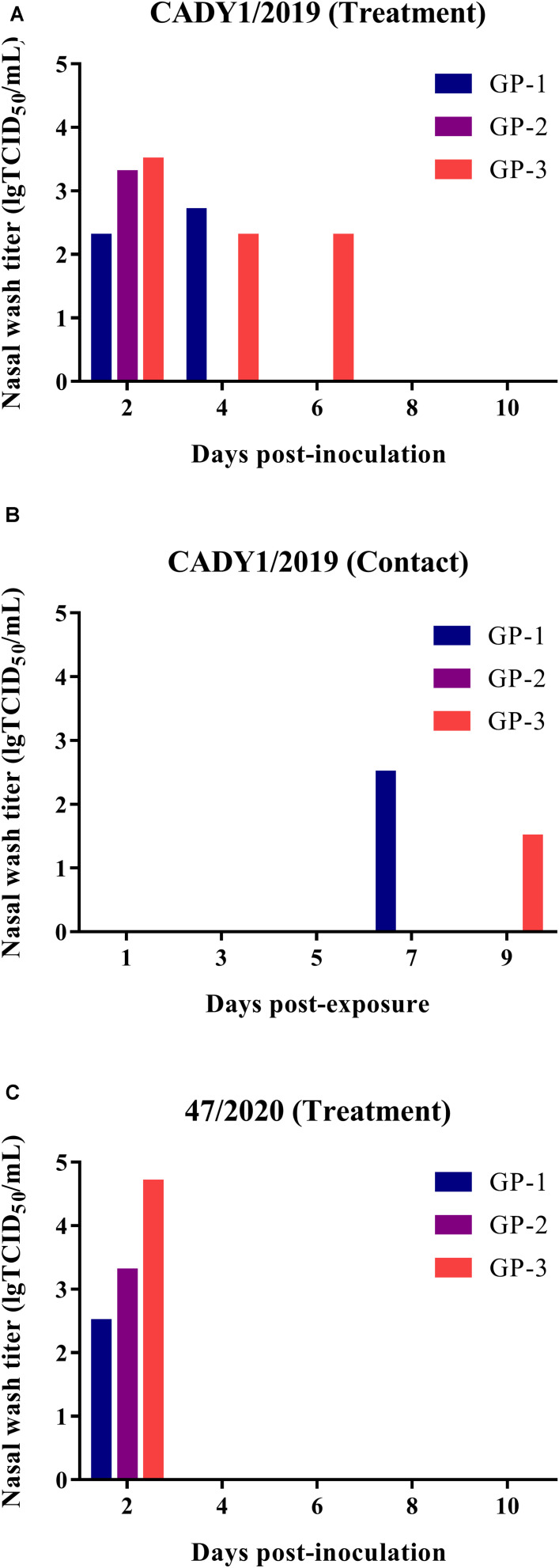
Replication and transmission of viruses in guinea pigs. Six guinea pigs were inoculated intranasally at a dose of 10^6^ TCID_50_ of CADY1/2019 or 47/2020 in 300 μL and were then co-housed with three naïve guinea pigs at 1 dpi. Three guinea pigs were intranasally inoculated with 300 μL PBS as control group. The nasal washes of the guinea pigs were collected at 2, 4, 6, 8, 10 dpi for virus titration in MDCK cells. The sera of guinea pigs were collected at 21 dpi for HI titers detection. **(A)** The nasal wash titers of CADY1/2019 treatment group. **(B)** The nasal wash titers of CADY1/2019 contact group. **(C)** The nasal wash titers of 47/2020 treatment group.

To detect the transmission of CADY1/2019, three guinea pigs were inoculated intranasally with 10^6^ TCID_50_ of the virus. At 1 dpi, three naïve guinea pigs were co-housed with treatment guinea pigs in the same cage. Nasal washes were collected at 2, 4, 6, 8, and 10 dpi, and viral titers were detected in MDCK cells. Guinea pigs were necropsied at 21 dpi, and HI titers were detected by HI and MN assays. In the CADY1/2019 inoculation group, virus shedding (with titers of 2.30–3.50 lgTCID_50_/mL) was detected at 2–6 dpi (Figure 6A). In the CADY1/2019 contact group, virus shedding was detected at 7 and 9 days post-exposure (dpe), and the viral titers were 1.50–2.50 lgTCID_50_/mL (Figure 6B). Three treatment guinea pigs seroconverted and HI titers were 1:1,029–1:2,048. Two of three guineas pigs exposed to treatment guineas pigs seroconverted and HI titers were 1:64 and 1:256 (Supplementary Table S2). In the 47/2020 inoculation group, viral shedding with titers of 2.50–4.70 lgTCID_50_/mL was detected at 2 dpi (Figure 6C). The inoculation guinea pigs seroconverted and HI titers were 1:512–1:1,024 (Supplementary Table S2). Viral shedding and seroconversion were not detected in the contact group of 47/2020 and control guinea pigs. These results indicate that CADY1/2019 replicated in the respiratory tract and efficiently transmitted among guinea pigs through direct contact.

## Discussion

Canine are documented as a reservoir of IAV since 2004 (Crawford et al., 2005). That diverse subtype of IAVs that have emerged in canine increases the concern about canine serving as a potential source of epidemic or pandemic viruses. Here we found that CADY1/2019, an avian-origin canine influenza virus, posed a potential to infect human and other mammals. CADY1/2019 bound to both avian-type and human-type receptors. The antigenicity of CADY1/2019 was obviously different from current human H3N2 influenza viruses or recommended vaccine strains. CADY1/2019 has similar fusion pH values as human influenza viruses and effectively replicates in canine, human, and swine cells. CADY1/2019 replicated in the respiratory tract of guinea pigs and effectively transmitted among them through direct physical contact.

In China, avian-origin H3N2 CIV infection in pet dog was traced to 2006 (Li et al., 2010). H3N2 CIVs circulate in pet dogs, farmed dogs, and running dogs in many provinces of China, of which the majority are of avian-origin (Li et al., 2010; Lin et al., 2012; Su et al., 2013; Yang et al., 2014; Shen et al., 2020). The similarity index values of avian-origin H3N2 CIVs were higher than those of AIVs, and these indicated that H3N2 CIVs acquired adaptation in canine (Li et al., 2018). A novel antigenic and genetic distinct clade of H3N2 CIVs was reported in China in 2016. The novel H3N2 CIVs that acquired an adaptation mutation to infect mammals, replaced previous CIVs and posed a zoonotic potential (Lyu et al., 2019). CADY1/2019 belonged to such a clade. Besides, few CIVs fall into human/swine lineage, such as Guangxi isolates (Chen et al., 2015), which indicates that those viruses might originate from swine or human influenza viruses. CIVs have become increasingly diverse (Song et al., 2012; Moon et al., 2015; Na et al., 2015; Chen et al., 2018; Lyu et al., 2019; Shen et al., 2020) and have zoonotic potential.

Receptor binding specificity is one of the key factors involving in interspecies transmission (Shi et al., 2014). Previous study showed that CIV HAs exhibited an avian receptor-binding preference. According to the glycan-binding analyses, CIV HA revealed a strong binding preference for the α2–3-linked SAs and mixed α2–3/α2–6 branched SAs (Pulit-Penaloza et al., 2017). A/canine/Illinois/41915/2015 preferentially bound avian α2,3-linked SA receptor (Martinez-Sobrido et al., 2020). Our result showed that CADY1/2019 both bound to avian-type and human-type receptors. The V214I and S159N mutations in the HA protein enhanced the binding affinity of H5N1 IAV for α-2,6 SA (Wang et al., 2010; Watanabe et al., 2011). That mutations were detected in the CADY1/2019 virus, which might contribute to the binding affinity for the α-2,3-linked and α-2,6-linked sialic acid receptor. The binding affinity for the α-2,3-linked and α-2,6-linked sialic acid receptor of CADY1/2019 indicated the potential risk to infect human and other mammals.

HA activation pH values are related to host adaptation, pandemic potential, and cross-species transmission (Reed et al., 2010; Imai et al., 2012; Galloway et al., 2013; Zaraket et al., 2013; Russell et al., 2018). Generally, HA activation pH values of AIVs were higher than those of human-adapted IAVs (Reed et al., 2010; Zaraket et al., 2013). Relatively unstable HA proteins (activation pH 5.6–6.0) were essential for efficient replication and transmission of H5N1 AIVs among avian species. However, relatively stable HA proteins (activation pH < 5.6) were crucial for H5N1 AIVs to efficiently replicate and transmit by airborne in ferrets (Reed et al., 2010; DuBois et al., 2011; Imai et al., 2012; Linster et al., 2014). Similarly, a relatively stable HA protein (activation pH ≤ 5.5) is essential for pdm/09 (Russier et al., 2016). H1N1, H2N2, and H3N2 viruses pose relatively stable HA proteins (activation pH 5.0–5.4) (Scholtissek, 1985; Galloway et al., 2013; Costello et al., 2015). Our result shows that CADY1/2019 is a relatively stable HA protein with an activation pH of 5.5, which is 0.1 units higher than that of 47/2020, a H3N2 virus currently circulating in humans. These fusion values are similar to those of two American CIVs (Martinez-Sobrido et al., 2020). Do H3N2 CIVs pose similar activation pH values? Further studies will be needed to address this question. The S221P mutation in the HA protein of H5N1 IAV reduced HA activation pH by 0.2 units (DuBois et al., 2011). CADY1/2019 poses proline (P) at position 221 in the HA protein, which might contribute to the relative stable HA protein. The pH is acidic (pH5.5–6.9) in the respiratory tract of mammals and nasal pH drop to 5.2 upon infection with influenza viruses (Hoekstra and Klappe, 1993; Washington et al., 2000; Fischer and Widdicombe, 2006; Galloway et al., 2013). The HA activation/inactivation pH values of CADY1/2019 are similar to those of human-adapted influenza viruses. Relatively stable HA proteins might favor CADY1/2019 to infect human and other mammals.

Antigenic drift caused by single or multiple mutations in HA protein is essential for IAV to escape the host immune system. The mutations at position 145, 155, 156, 158, 159, 189, and 193 in HA protein of human H3N2 seasonal influenza viruses were responsible for the antigenic phenotype (Koel et al., 2013). Compared to the vaccine candidate strain recommended by the WHO for northern hemisphere during 2017–2021 and human H3N2 viruses currently circulating in southern China, CADY1/2019 mutated at positions 145, 156, 158, 159, and 189. The HI and MN results indicated that guinea pigs sera against CADY1/2019 did not react with human H3N2 virus 47/2020 or 1194/2019, vice versa. Previous studies showed the antigenicity of CIVs was different from human viruses (Pulit-Penaloza et al., 2017; Martinez-Sobrido et al., 2020). Our results were consistent with previous studies and indicated that the antigenicity of CADY1/2019 was obviously different from those of current human viruses. CADY1/2019 poses a potential threat to humans because we lack immunity against such CIVs.

Multiple-genetic mutations contribute to polymerase activity and replication of IAVs. The W222L mutation in HA protein increased infectivity and replication of H3N2 CIV in mammalian cells (Yang et al., 2013). The K389R mutation in the PB2 protein enhanced the polymerase activity and replication of H7N9 IAV in human and mammalian cells (Hu et al., 2017). The D3V mutation in the PB1 protein increased polymerase activity and viral replication of H5N1 IAV in avian and mammalian cells (Elgendy et al., 2017). The N409S substitution in PA protein increased polymerase activity and replication of H7N9 IAV in A549 cells (Yamayoshi et al., 2014). CADY1/2019 carries the above mutations. H3N2 CIV reached the viral peak titer of 7.7 lgTCID_50_/mL in MDCK cells at 24 hpi (Tao et al., 2019). CADY1/2019 achieved a peak titer of 9.1 lgTCID_50_/mL in MDCK cells at 48 hpi. H3N2 CIV infection has already been confirmed in a human (Lyu et al., 2019). The H3N2 CIV isolated in America had limited replication in the lungs of swine (Abente et al., 2016). CADY1/2019 effectively replicated in HBE, A549, 3D/21, ST, and PK-15 cells. These findings indicated that CADY1/2019 exhibit higher replication capability and has the potential to infect humans and swine.

Guinea pigs were considered as an effective animal model for testing infection and pathogenesis of avian-origin CIVs. Viral shedding failed to be detected from contact guinea pigs when co-caged with guinea pigs intranasally infected with A/canine/Korea/01/2007 (H3N2) at a dose of 10^7⋅1^ EID_50_ (Lyoo et al., 2015). In the current study, virus shedding was detectable in two of three contact guinea pigs that co-caged with guinea pigs intranasally inoculated with CADY1/2019 at a dose of 10^6^ TCID_50_. Compared to 47/2020, a current human H3N2 virus, CADY1/2019 exhibited a longer viral shedding period and contact transmission ability in guinea pigs. Although seroconvesion was detected in the guinea pigs inoculated with 47/2020, virus replication in tissues of guinea pigs failed to be detected at 3 dpi. The virus shedding in guinea pigs inoculated with 47/2020 was only detected at 2 dpi. The short time of viral replication might account for undetectable viral titer of 47/2020 in tissues at 3 dpi. Compared to 47/2020, CADY1/2019 replicated efficiently in both upper and lower respiratory tract of guinea pigs. The following amino acids increased the replication and virulence of AIVs in mammalian hosts: 89V and 309D in PB2; 622G in PB1; 66S in PB1-F2; 224S in PA; 42S, 106M, 103F, and 106M in NS1; and 30D and 215A in M1 (Conenello et al., 2007; Jiao et al., 2008; Fan et al., 2009; Li et al., 2009; Schmolke et al., 2011; Spesock et al., 2011; Ayllon et al., 2014; Sun et al., 2014; Yamayoshi et al., 2014; Feng et al., 2016). CADY1/2019 has molecular characteristics that might account for its efficient replication and transmission in guinea pigs.

In conclusion, CADY1/2019, which has an antigenicity distinct from current human H3N2 IAVs or recommend vaccine strains, bound to avian-type receptor and human-type receptor, efficiently replicated in canine, human, and swine cells, caused direct-contact transmission among guinea pigs, and thus poses a potential threat to public health. There is now an urgent need for strengthening surveillance of CIVs and monitoring their pathogenicity.

## Data Availability Statement

The datasets presented in this study can be found in online repositories. The names of the repository/repositories and accession number(s) can be found in the article/Supplementary Material.

## Ethics Statement

The animal study was reviewed and approved by the Experimental Animal Administration and Ethics Committee of South China Agriculture University.

## Author Contributions

HS designed the experiments, analyzed the data, and wrote the manuscript. MW conducted the experiments and wrote the manuscript. RS and JW isolated viruses and revised manuscript. YG, YY, SL, HPS, LP, XC, XZ, QY, YWL, FX, MLL, YL, and XQ conducted the experiments. ML designed the experiments and revised manuscript. All authors contributed to the article and approved the submitted version.

## Conflict of Interest

The authors declare that the research was conducted in the absence of any commercial or financial relationships that could be construed as a potential conflict of interest.
