# Supplementary material for: Molecular Characteristics, Antigenicity, Pathogenicity, and Zoonotic Potential of a H3N2 Canine Influenza Virus Currently Circulating in South China
Source: Front Microbiol. 2021 Mar 9;12:628979. doi: 10.3389/fmicb.2021.628979 (PMC7985081; doi:10.3389/fmicb.2021.628979)
Supplement: Supplementary file 1 [file Data_Sheet_1.docx]

Supplementary Material

**Supplementary tables**

**Table S1.** Genes of CADY1/2019 with the closest nucleotide homology sequence in GenBank.

| **Gene** | **Virus with the highest identity** | **Identity (%)** | **Accession No.** |
| --- | --- | --- | --- |
| PB2 | A/canine/Guangdong/3/2018(H3N2) | 99.56 | MK120001.1 |
| PB1 | A/canine/Guangdong/3/2018(H3N2) | 99.82 | MK119998.1 |
| PA | A/canine/Guangdong/3/2018(H3N2) | 99.49 | MK119995.1 |
| HA | A/canine/Guangdong/3/2018(H3N2) | 99.12 | MK119983.1 |
| NP | A/canine/Guangdong/3/2018(H3N2) | 99.87 | MK119992.1 |
| NA | A/canine/Guangdong/3/2018(H3N2) | 99.57 | MK119986.1 |
| M | A/canine/Guangdong/3/2018(H3N2) | 99.80 | MK120004.1 |
| NS | A/canine/China/Shanghai-0103-2045/2019(H3N2) | 99.40 | MK758033.1 |
|  | A/canine/Guangdong/2/2018(H3N2) | 99.40 | MK119988.1 |

**Table S2.** HI titers of sera collected from guinea pigs

| **Virus** | **Treatment** | |  | | | **Contact** | | |
| --- | --- | --- | --- | --- | --- | --- | --- | --- |
|  | **GP1^a^** | **GP2** | | **GP3** |  | **GP1** | **GP2** | **GP3** |
| CADY1/2019 | 1626 | 1290 | | 2048 |  | 256 | <10 | 64 |
| 47/2020 | 512 | 1024 | | 512 |  | <10 | <10 | <10 |
| PBS | <10 | <10 | | <10 |  | <10 | <10 | <10 |

^a^ GP means guinea pig; HI titers were expressed at geometric mean titer.

**Supplementary figures**

**Figure S1.** Phylogenetic analysis of PB2 gene of CADY1/2019. Phylogenetic trees for whole gene segments of CADY1/2019 created with 1000 bootstrap replicates using MEGA 7.0 software. Solid red square indicated CADY1/2019 in each phylogenetic tree. Phylogenetic trees of PB2 genes based on nt 28 to 2307.

**Figure S2.** Phylogenetic analysis of PB1 gene of CADY1/2019. Phylogenetic trees for whole gene segments of CADY1/2019 created with 1000 bootstrap replicates using MEGA 7.0 software. Solid red square indicated CADY1/2019 in each phylogenetic tree. Phylogenetic trees of PB1 genes based on nt 25 to 2298.

**Figure S3.** Phylogenetic analysis of PA gene of CADY1/2019. Phylogenetic trees for whole gene segments of CADY1/2019 created with 1000 bootstrap replicates using MEGA 7.0 software. Solid red square indicated CADY1/2019 in each phylogenetic tree. Phylogenetic trees of PA genes based on nt 25 to 2175.

**Figure S4.** Phylogenetic analysis of NP gene of CADY1/2019. Phylogenetic trees for whole gene segments of CADY1/2019 created with 1000 bootstrap replicates using MEGA 7.0 software. Solid red square indicated CADY1/2019 in each phylogenetic tree. Phylogenetic trees of NP genes based on nt 46 to 1542.

**Figure S5.** Phylogenetic analysis of M gene of CADY1/2019. Phylogenetic trees for whole gene segments of CADY1/2019 created with 1000 bootstrap replicates using MEGA 7.0 software. Solid red square indicated CADY1/2019 in each phylogenetic tree. Phylogenetic trees of M genes based on nt 26 to 1007.

**Figure S6.** Phylogenetic analysis of NS gene of CADY1/2019. Phylogenetic trees for whole gene segments of CADY1/2019 created with 1000 bootstrap replicates using MEGA 7.0 software. Solid red square indicated CADY1/2019 in each phylogenetic tree. Phylogenetic trees of NS genes based on nt 27 to 864.
