# Supplementary figures and images for: Molecular Characteristics, Antigenicity, Pathogenicity, and Zoonotic Potential of a H3N2 Canine Influenza Virus Currently Circulating in South China
Source: Front Microbiol. 2021 Mar 9;12:628979. doi: 10.3389/fmicb.2021.628979 (PMC7985081; doi:10.3389/fmicb.2021.628979)

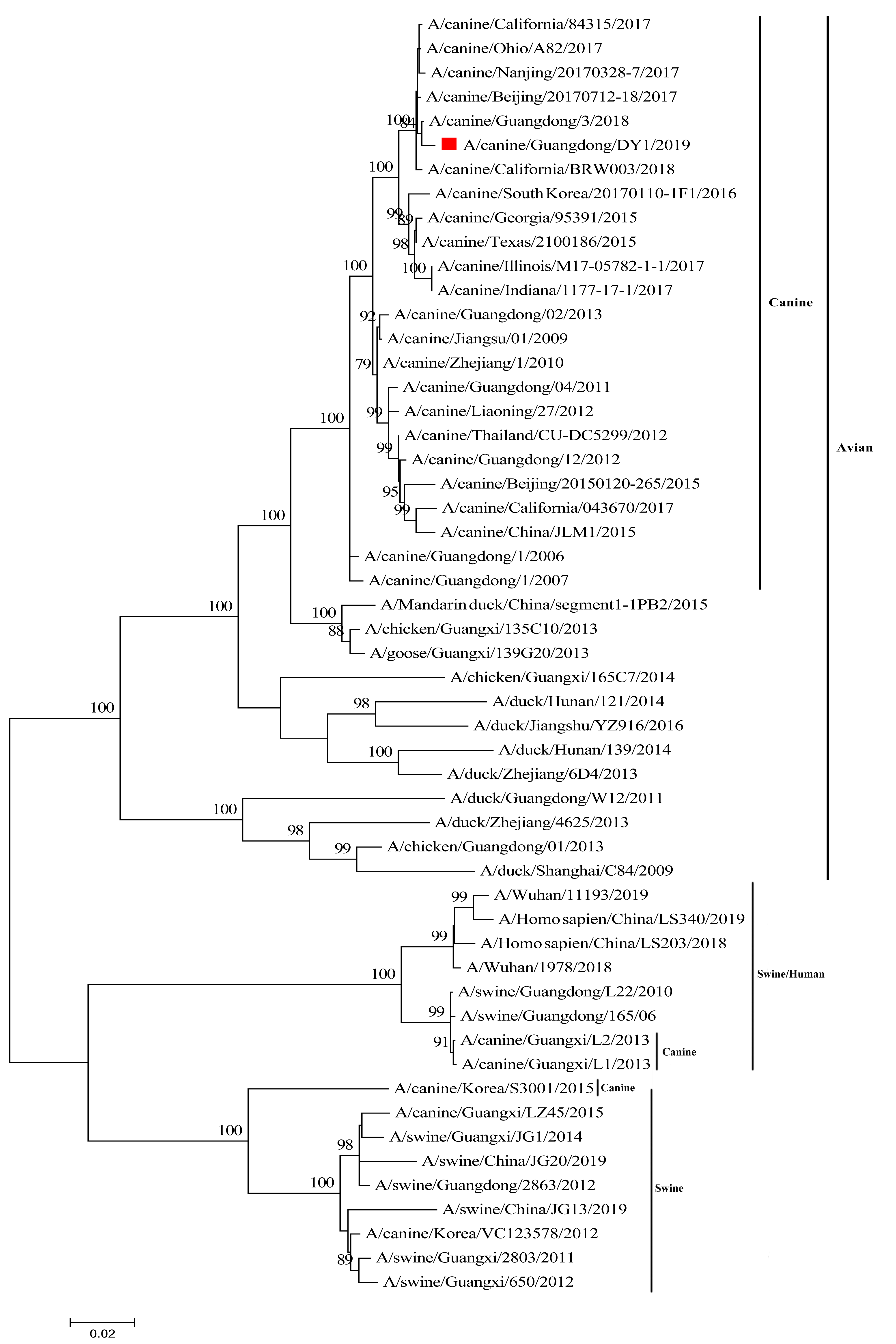

Supplement: Supplementary file 2 [file Image_1.TIF]

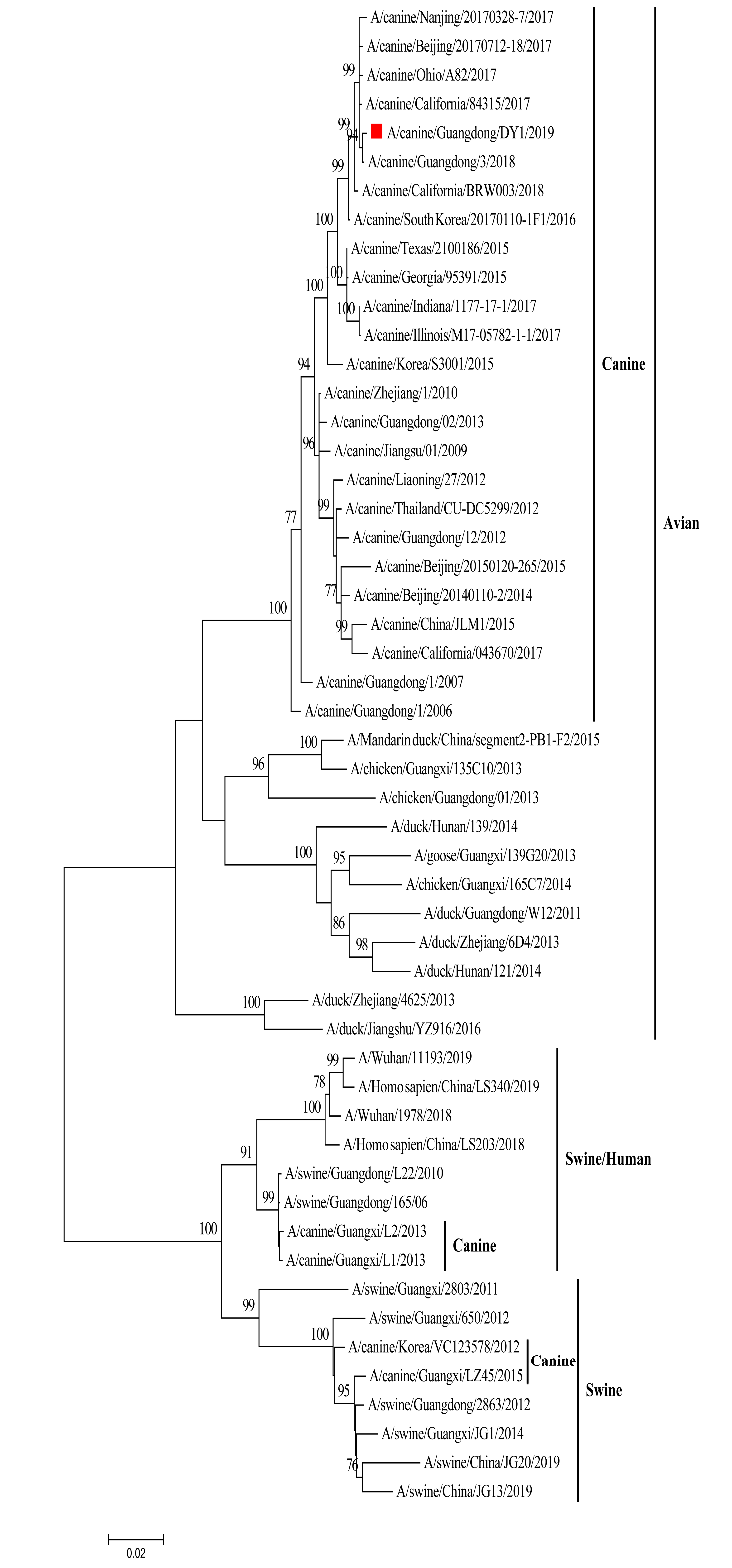

Supplement: Supplementary file 3 [file Image_2.TIF]

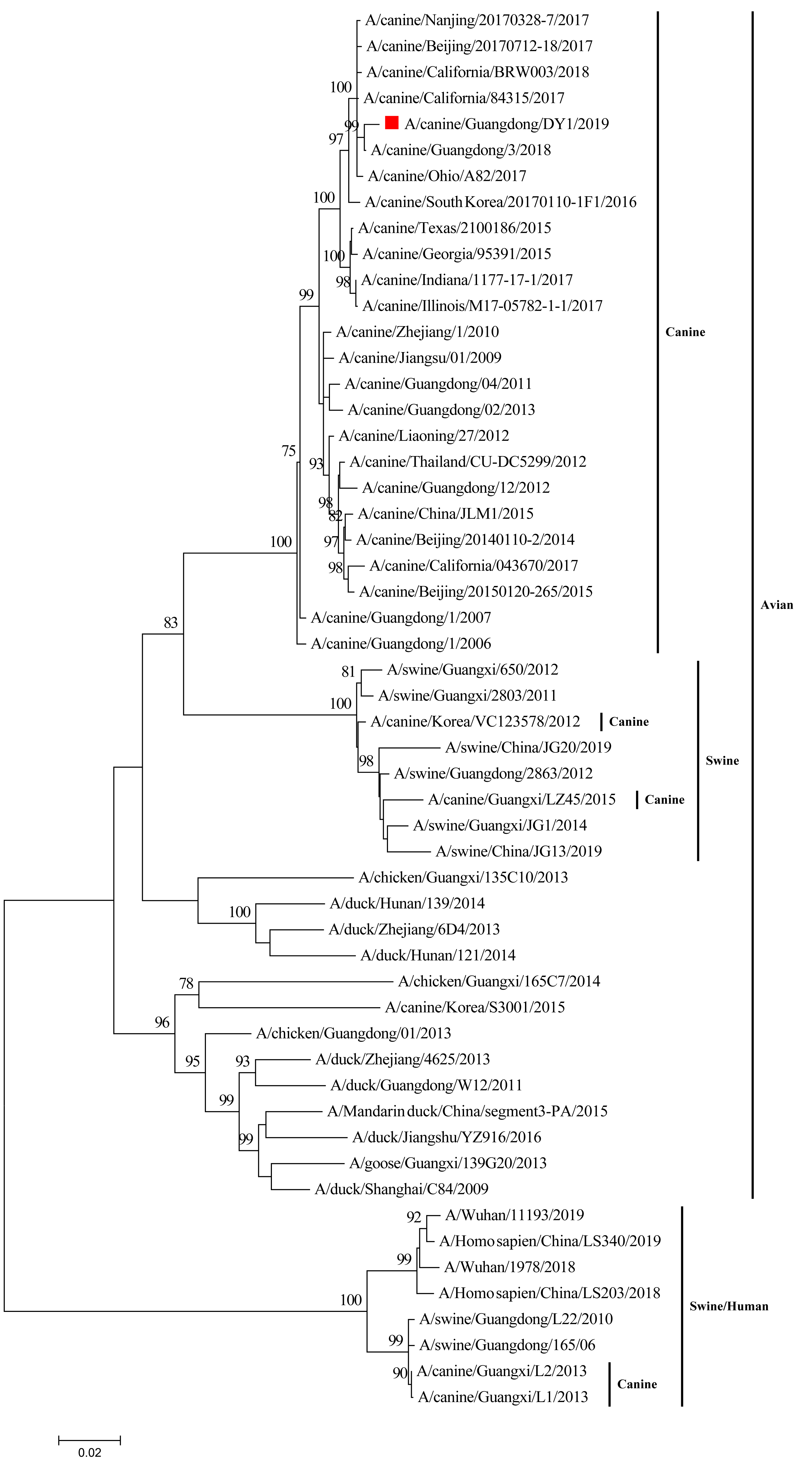

Supplement: Supplementary file 4 [file Image_3.TIF]

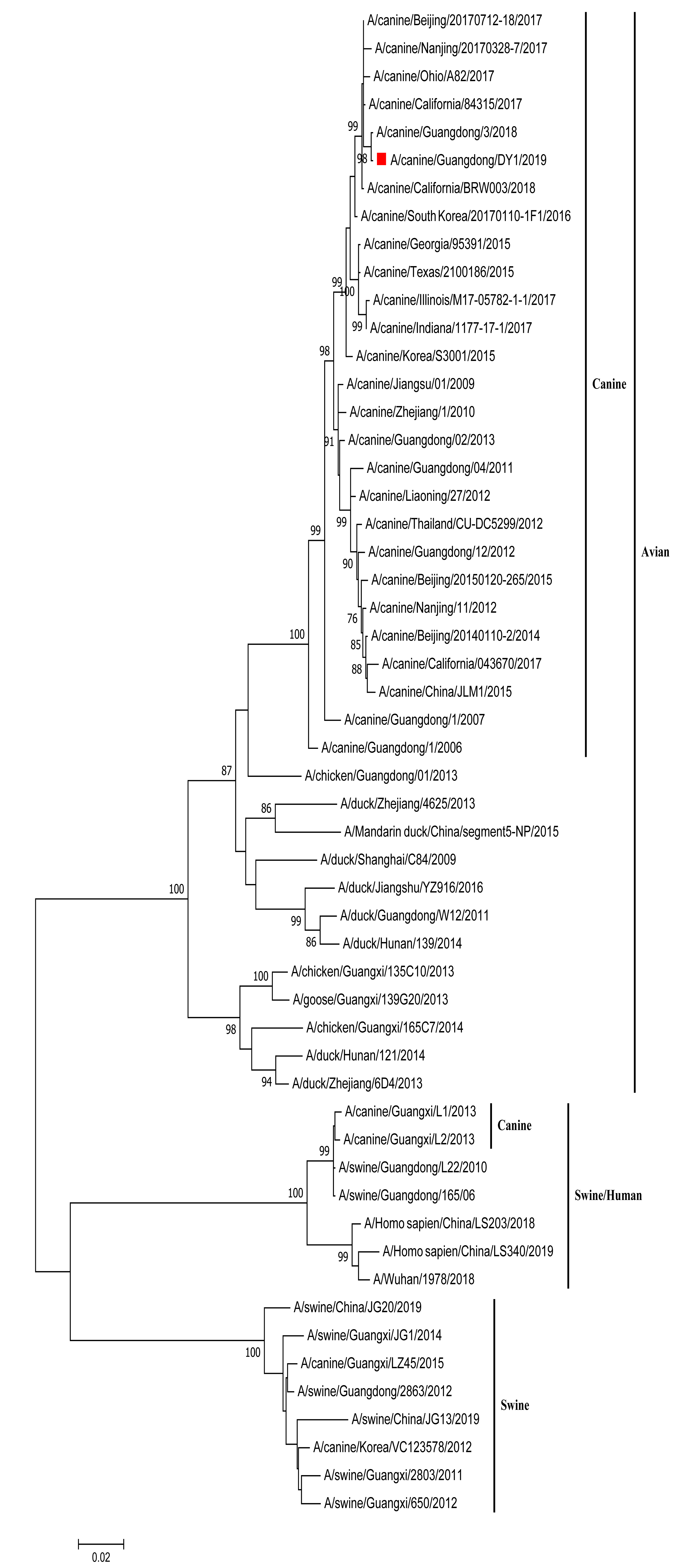

Supplement: Supplementary file 5 [file Image_4.TIF]

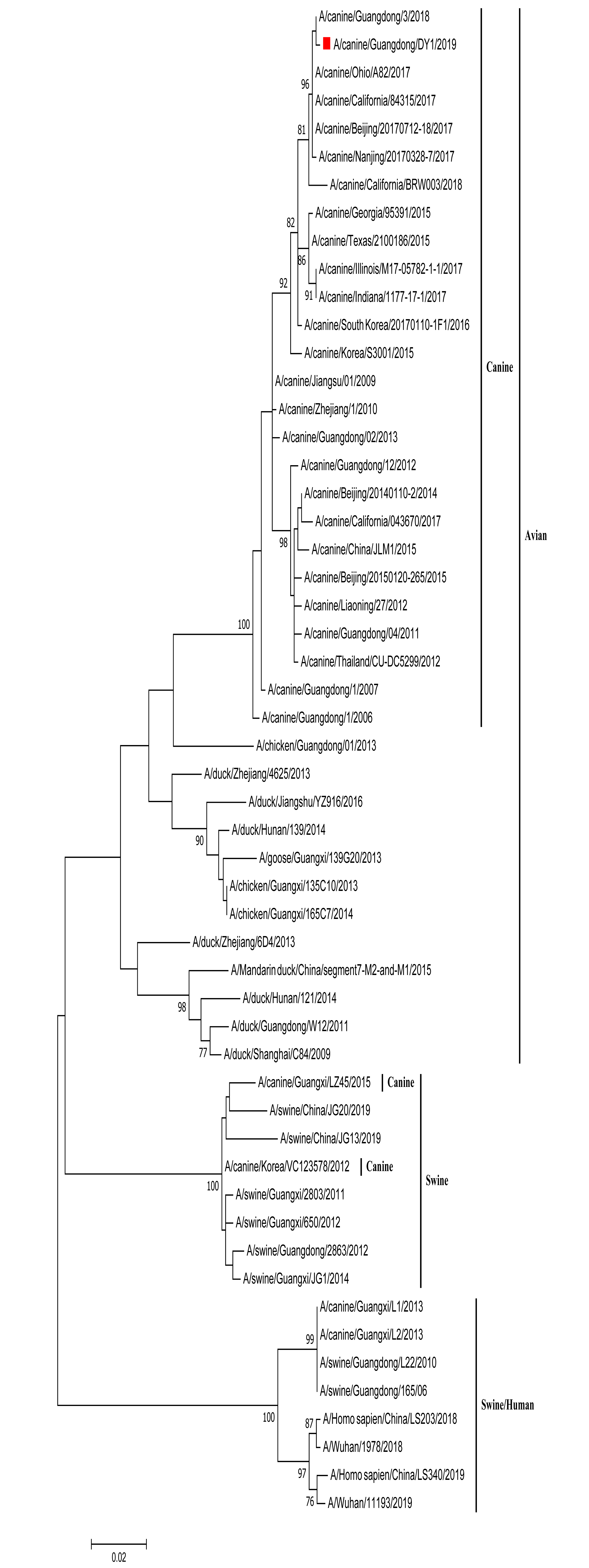

Supplement: Supplementary file 6 [file Image_5.TIF]

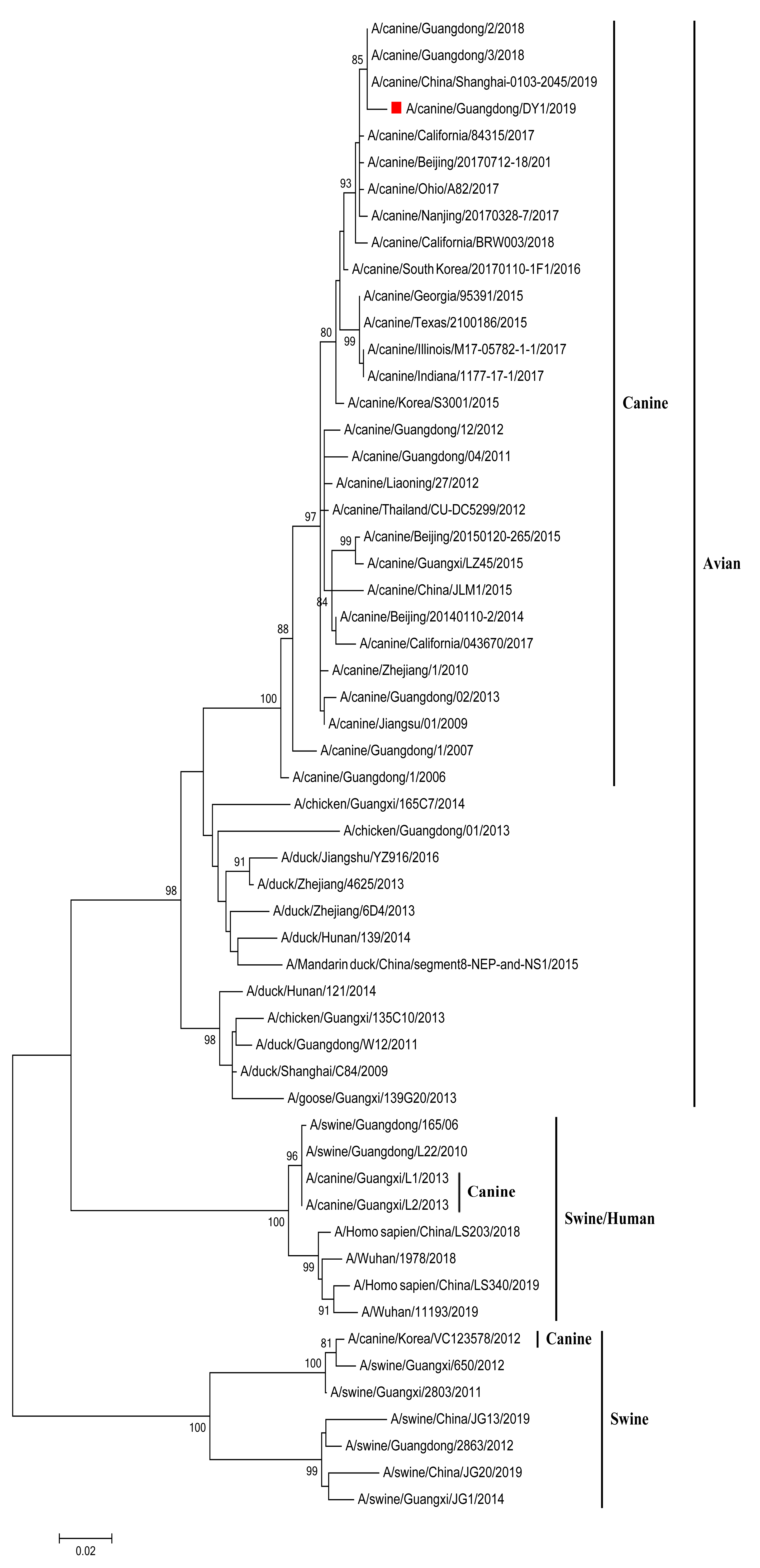

Supplement: Supplementary file 7 [file Image_6.TIF]
